# Role of Viral miRNAs and Epigenetic Modifications in Epstein-Barr Virus-Associated Gastric Carcinogenesis

**DOI:** 10.1155/2016/6021934

**Published:** 2016-02-10

**Authors:** Aldo Giudice, Giovanni D'Arena, Anna Crispo, Mario Felice Tecce, Flavia Nocerino, Maria Grimaldi, Emanuela Rotondo, Anna Maria D'Ursi, Mario Scrima, Massimiliano Galdiero, Gennaro Ciliberto, Mario Capunzo, Gianluigi Franci, Antonio Barbieri, Sabrina Bimonte, Maurizio Montella

**Affiliations:** ^1^Epidemiology Unit, National Cancer Institute of Naples “G. Pascale Foundation”, IRCCS, 80131 Naples, Italy; ^2^Department of Onco-Hematology, IRCCS, Cancer Referral Center of Basilicata, 85028 Rionero in Vulture, Italy; ^3^Department of Pharmacy, University of Salerno, Fisciano, 84084 Salerno, Italy; ^4^Department of Experimental Medicine II, University of Naples, 81055 Naples, Italy; ^5^Scientific Direction, National Cancer Institute “G. Pascale Foundation”, IRCCS, 80131 Naples, Italy; ^6^Department of Medicine and Surgery, University of Salerno, Baronissi, 84081 Salerno, Italy; ^7^Animal Facility Unit, National Cancer Institute of Naples “G. Pascale Foundation”, IRCCS, 80131 Naples, Italy; ^8^Division of Abdominal Surgical Oncology, Hepatobiliary Unit, National Cancer Institute “G. Pascale Foundation”, IRCCS, 80131 Naples, Italy

## Abstract

MicroRNAs are short (21–23 nucleotides), noncoding RNAs that typically silence posttranscriptional gene expression through interaction with target messenger RNAs. Currently, miRNAs have been identified in almost all studied multicellular eukaryotes in the plant and animal kingdoms. Additionally, recent studies reported that miRNAs can also be encoded by certain single-cell eukaryotes and by viruses. The vast majority of viral miRNAs are encoded by the herpesviruses family. These DNA viruses including Epstein-Barr virus encode their own miRNAs and/or manipulate the expression of cellular miRNAs to facilitate respective infection cycles. Modulation of the control pathways of miRNAs expression is often involved in the promotion of tumorigenesis through a specific cascade of transduction signals. Notably, latent infection with Epstein-Barr virus is considered liable of causing several types of malignancies, including the majority of gastric carcinoma cases detected worldwide. In this review, we describe the role of the Epstein-Barr virus in gastric carcinogenesis, summarizing the functions of the Epstein-Barr virus-encoded viral proteins and related epigenetic alterations as well as the roles of Epstein-Barr virus-encoded and virally modulated cellular miRNAs.

## 1. Introduction

The Epstein-Barr virus (EBV) was the first discovered human tumor-causing virus considered as the etiologic agent of Burkitt's lymphoma (BL), an unusual African pediatric lymphoma [1].

**Figure 1:**
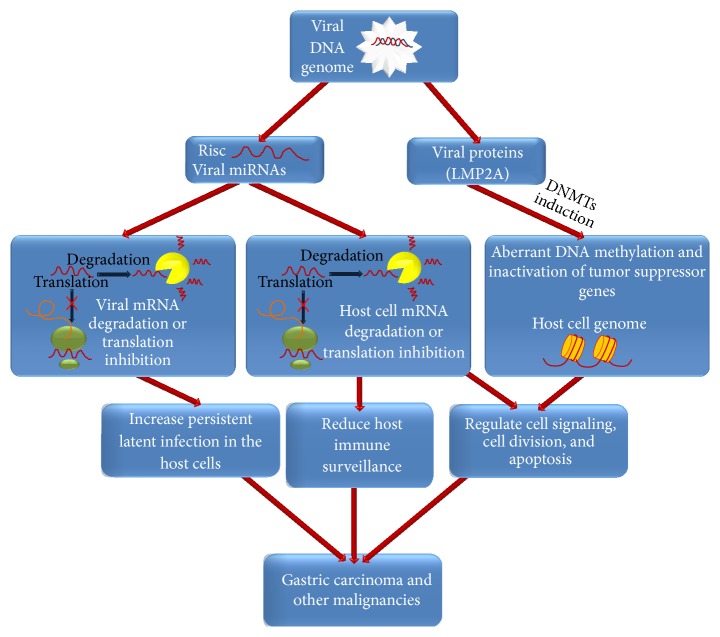
Graphical representation of possible mechanisms by which viral miRNAs and viral proteins might contribute to EBV-associated gastric carcinogenesis. This model indicates that EBV-encoded miRNAs (e.g., BART miRNAs and BHRF-1 miRNAs) target viral genes to mediate immune evasion or maintenance of latency, whereas some viral proteins (e.g., LMP2A) promote aberrant host DNA methylation and subsequent inactivation of tumor suppressor genes via DNA methyltransferases (DNMTs) induction. In addition, these viral miRNAs incorporated into RISC complex can also interact directly with specific host genes involved in immune surveillance, cell proliferation, and apoptosis, playing a crucial role in the aetiology of diverse diseases including EBVaGC.

Specifically, EBV is ubiquitous member of the human gamma-herpesvirus family that causes mononucleosis during acute and lytic infection and also establishes a persistent and latent infection in more than 90% of the human population. EBV latent infection has been demonstrated to be involved in multiple types of cancer that primarily develop in lymphocytes and epithelial cells. These include malignant tumors that develop in the immunocompromised conditions such as AIDS-associated lymphomas and posttransplant lymphoproliferative disease [2, 3] and also several human cancers that develop in the immunocompetent patients such as BL, Hodgkin's lymphoma, B-cell and T-cell lymphomas, epithelial nasopharyngeal carcinoma (NPC), and some forms of gastric carcinomas [4–6]. Gastric cancer is the fourth most common cancer in the world and the second leading cause of cancer-related death. Globally, gastric cancer poses a significant public health burden, both economically and socially [7, 8]. Risk factors of gastric cancer are multifactorial; hence genes, diet, age, and chronic inflammation need to be evaluated in connection with infectious agents (EBV,* Helicobacter pylori*) and environmental factors (e.g., alcohol and smoking) [8]. Notably, EBV-associated gastric carcinoma (EBVaGC) represents almost 10% of all gastric carcinoma cases and expresses restricted EBV latent genes (Latency I) [4, 9]. In recent years, it has become increasingly evident that EBV may contribute to gastric carcinogenesis through the expression of viral proteins and microRNAs (miRNAs) [10] (Figure 1). A growing body of scientific evidence also suggests that, in addition to genetic alterations, epigenetic alterations, including aberrant DNA methylation of CpG islands and posttranslational modifications of histones, are involved in the development and progression of EBVaGC [10, 11]. This review briefly summarizes remarkable advancements in our understanding of the functions and mechanisms of action of herpesviral miRNAs in gastric carcinoma in recent years. In particular it discusses how the expression of viral proteins and epigenetic alterations contribute to EBVaGC and the roles of EBV-encoded and virally modulated cellular miRNAs in the respective viral life cycles and in EBV-associated gastric carcinoma.

## 2. Biogenesis of EBV-Encoded miRNAs

The generation of viral miRNA and selection of targets are totally dependent on the host molecular miRNA apparatus involved in the maturation and silencing. Most viruses utilize a strategy similar to that of the host cell to produce the viral pri-miRNAs by using the host RNA polymerase II (RNAP II) [12, 13]. Exceptionally, certain viral miRNAs (such as those from mouse *γ*-herpesvirus 68, MHV68) are produced from tRNA-like genes transcribed by host RNA polymerase III (RNAP III) and processed Drosha independently by host tRNase Z [14]. A minority of viruses from the herpesvirus and retrovirus families also utilize noncanonical pathways other than “tRNA-like” to generate pre-miRNA molecules [15–22]. For instance, herpesvirus saimiri (HVS), an oncogenic *γ*-herpesvirus that infects New World monkeys, expresses small nuclear RNAs (snRNAs) of the Sm-class called HSURs which are processed by the integrator complex to produce viral pre-miRNAs. Subsequently, both the MHV68 and HVS pre-miRNAs are processed by Dicer to generate miRNAs [23]. Additionally, some retroviruses such as foamy viruses (FVs) and bovine leukemia virus (BLV) are able to express pri-miRNAs via RNAP III [18, 19, 21]. While some retroviral pri-miRNAs are processed by Drosha in the frame of RNAP III transcripts, some others may be directly processed by Dicer bypassing Drosha processing. Production of retroviral miRNAs without having recourse to Drosha-mediated cleavage of the RNA genome intermediate has been found to represent an important biogenesis strategy that may eventually take an active part in reducing overall viral fitness [18, 19, 21]. In brief, viral miRNA biogenesis initiates in the nucleus, where after transcription by host RNA polymerase II the microprocessor complex which contains the host RNase III endonuclease Drosha and its interaction partner DGCR8 (also known as Pasha in Drosophila) [24] cleaves pri-miRNA hairpin structure to pre-miRNAs. The vast majority of pri-miRNAs contain approximately 80 nucleotide hairpin secondary structures that can be intronic or exonic. Approximately 60 pre-miRNA nucleotides are liberated and rapidly exported from the nucleus to the cytoplasm by exportin-5/Ran GTPase pathway. Once in the cytoplasm, they are further processed by a second host RNase III endonuclease, Dicer, into a short double-stranded (ds) RNA or RNA duplex. The strand guide of these mature miRNAs of approximately 22 nucleotides is then incorporated into a protein complex known as the RNA-induced silencing complex (RISC), while the other strand called the passenger is rapidly degraded [13, 25]. RISC complex can incorporate both strands of miRNAs generated in EBV-infected cells. The major component of RISC is the Argonaute (Ago) protein. In mammalian cells there are four Ago proteins which are all incorporated into RISC; however, only Ago2 shows endonuclease activity. Binding of Ago-loaded miRNAs (miRISC) to a mRNA bearing extensive sequence complementary to miRNA generally results in mRNA cleavage and degradation, whereas binding to mRNAs bearing partial complementarity to miRNA results mainly in translational arrest [12, 13, 26]. There is no proof that any animal virus encodes additional miRNA-processing factors or RISC components. Therefore, viral miRNAs biogenesis largely relies on host-derived enzymes or proteins. Interestingly, EBV miRNAs can be subdivided into two groups, Bam HI fragment H rightward open reading frame 1 microRNAs (BHRF1 miRNAs) and Bam HI-A rightward transcripts microRNAs (BART miRNAs), based on their locations [27, 28]. Specifically, BHRF1 miRNAs are located within introns of the BHRF1 gene and generated from the long EBNA transcripts; whereas BART miRNAs are instead located in introns included within the BART transcripts [27, 28]. Nevertheless, how EBV miRNA expression is finely regulated remains largely unknown. Various studies suggest that BART transcripts/miRNAs are transcribed from P(1) and P(2) promoters in both B-cells and epithelial cells [29]. The BART promoter region contains putative binding sites for diverse transcription factors. Among transcription factors, C/EBP*β* was reported to positively modulate the expression of BART [30]. Notably, expression of the EBV miRNAs was detected in several human cancer cell lines; in fact, expression of the EBV miRNAs was observed in EBVaGC [31] and peripheral T-cell lymphoma [32] besides being shown in B-cell lines and nasopharyngeal carcinoma EBV-infected cells [27, 28, 33]. Most plant miRNAs as well as some rare viral miRNAs bind to their targets with perfect complementarity even though this is generally uncommon in higher order animals. Once bound to the target, miRNAs behave similar to siRNAs, inducing specific and irreversible endonucleolytic cleavage event in the target transcripts [34]. The question why this mode of miRNA-mediated action is so rarely used by animal host miRNAs is puzzling [26].

## 3. Mechanism of EBV Infection, Viral Proteins Expression Profile during Latency, and EBV-Associated Gastric Carcinogenesis

Mounting scientific evidence describes herpesviruses having two distinct life cycles: lytic replication and latency. Notably, EBV may infect host gastric epithelial cells through direct and indirect mechanisms. In the direct infection, viral glycoproteins attach to cellular receptors and drive viral proteins conformational changes that promote fusion of the viral envelope with the epithelial cell membrane [35]. In the indirect infection, instead, EBV initially infects B lymphocytes. Subsequently, EBV-infected B-cells, through direct cell-to-cell contact, infect epithelial cells via the capped adhesion molecules [36]. Following primary infection, EBV, after an early replication phase, establishes persistent latent infection in the host cells. During latency, viral genomes exist as extrachromosomal episomes in the nucleus and, being largely silenced by host-driven methylation of CpG island motifs, are only able to express a small subset of genes including latent proteins with oncogenic potential and viral miRNAs [36].

As a result, the latency enables the virus to efficiently evade the host immune response causing persistent infections over a lifetime. This is a feature common to all herpesviruses [4, 37]. Considering the subset of viral genes expressed, herpesviruses-associated tumors have been subdivided into four types: latency Ia, latency Ib, latency II, and latency III. EBVaGC belongs to latency type I, where the viral genes EBV nuclear antigen 1 (EBNA1), latent membrane protein 2A (LMP2A), Bam HI-A rightward transcripts (BARTs), and EBV-encoded small RNA (EBER1/2) may be expressed [10, 38, 39]. In particular, the expression of latency genes fulfils a relevant task in the initiation and neoplastic progression of EBV-associated epithelial cancers including EBVaGC. For instance, the viral protein EBNA1 is absolutely required in maintaining and replicating the viral episomal genome by the host cell DNA polymerase machinery in all EBV-associated malignancies. EBNA1 may contribute to cell survival after DNA damage and to genetic instability in EBV-infected epithelial cells [40].

Specifically, in both NPC and EBVaGC, EBNA1 can promote the decline of the promyelocytic leukemia (PML) nuclear bodies, including many regulatory proteins, by binding and modulating casein kinase 2 (CK2) or ubiquitin-specific protease 7, which in turn degrades p53 [41]. Therefore, EBNA1 may increase the genetic instability of the EBV-infected epithelial cells and facilitate the oncogenesis mainly by decreasing p53 levels following DNA damage. Moreover, EBNA1 may also participate in tumor development by suppressing transforming growth factor-beta (TGF-*β*) [42] and improving the nuclear factor-kappa B (NF-*κ*B) signaling [43]. In addition to EBNA1, half of all EBVaGCs also express LMP2A, and therefore EBV latency configurations should be classified into Ia or Ib based on the presence or absence of LMP2A [38, 44]. LMP2A viral protein, therefore, plays a paramount role in the transformation of epithelial cells by the induction and maintenance of tumor phenotypes. These activities range from resistance to apoptosis to cell proliferation, invasion, motility, and angiogenesis [45]. LMP2A also inhibits apoptosis by upregulation of the cellular survivin gene through the NF-*κ*B pathway [46]. In epithelial cells, LMP2A may activate not only the phosphatidylinositol 3-kinase (PI3K)/protein kinase B (PKB) pathway and phosphorylation of glycogen synthase kinase-3 but also the *β*-catenin signaling pathway. These molecular mechanisms induce several remarkable phenotypic changes including anchorage-independent growth and inhibition of differentiation [47–49]. These data herein reviewed collectively suggest that LMP2A viral protein not only has an impact on the NF-*κ*B pathway which upregulates survivin gene, therefore inhibiting apoptosis, but also promotes the induction of cancer stem cells in EBV-associated epithelial cancers.

## 4. Role of Epigenetic Alterations in EBV-Associated Gastric Carcinogenesis

The term epigenetics currently refers to the inheritable changes in gene expression which occur without an alteration of the genome at the level of nucleotide sequences. Epigenetic mechanisms are necessary to support the physiological organ growth and development and moreover to insure normal gene expression in different tissues [50–52]. However, nowadays, gastric carcinogenesis processes can be explained not only by genetic modifications [53–56] but also by epigenetic alterations such as DNA methylation, histone modifications, and noncoding RNAs [51, 57]. These modifications are part of the “Epigenetic code” and are essential to regulate the normal development and maintenance of tissue-specific gene expression in different mammalian cell types [51, 52]. Increasing evidence suggests that some environmental factors such as aging, diet, physical activity, chronic inflammation, and microbial infection can also affect gene methylation in gastric epithelia and promote the development of gastric cancer [58, 59]. Specifically, EBV infection was reported as a cause of increased methylation and repression of tumor suppressor genes in MKN7, a low methylation GC cell line [11]. Further studies also confirmed that abnormal DNA methylation in the promoter regions of the gene, which provides inactivation of tumor suppressor and other cancer-related genes, is the most well-defined epigenetic characteristic in EBVaGC but not in EBV nonassociated GC (EBVnGC) [11, 60–66]. Specifically, hypermethylation of tumor suppressor genes such as E-cadherin (CDH1), p14, p15, p16, p73, adenomatous polyposis coli (APC), and phosphatase and tensin homolog (PTEN) were observed in EBVaGC but not in EBVnGC [67, 68]. In addition, Hino et al. [46] also demonstrated that LMP2A induced the expression of phosphorylated signal transducer and activator of transcription 3 (pSTAT3), which stimulated the upregulation of DNA methyltransferases (DNMT) DNMT1 and DNMT2 in EBV-infected GC cells [69]. Recent studies by Zhao et al. [60] also reported that hundreds of genes implicated in cancer signaling pathways such as mitogen-activated protein kinase signaling, wnt signaling pathway, and cell adhesion molecules were hypermethylated following EBV infection [60]. The same authors also suggested that EBV infection induced aberrant CpG hypermethylation of several genes by upregulation of DNMT3b through LMP2A in EBV-positive AGS cells compared to EBV-negative AGS cells. Another important cellular alteration in EBVaGC is its resistance to programmed cell death (apoptosis). The frequency of apoptosis is markedly reduced in EBVaGC compared to EBVnGC [70]. Some studies hypothesize that both genes, somatostatin receptor 1 (SSTR1) and glutathione S-transferase P1 (GSTP1), are frequently hypermethylated in GC infected EBV tissues and modulate cell migration, proliferation, and apoptosis [67, 68, 71, 72]. However, the molecular mechanism of the aberrant DNA methylation following EBV infection is still unclear. As mentioned above, one possible mechanism is EBV induction of LMP2A overexpression which promotes STAT3 phosphorylation, further inducing DNMTs expression [60, 69]. Therefore, LMP2A may play an important role in cellular epigenetic dysregulation involved in the development and maintenance of EBV-associated cancer by increasing DNMTs expression.

## 5. Role of EBV-Encoded miRNAs in Gastric Carcinogenesis

The identification of two miRNAs, miR-15a and miR-16a, located in a deletion region of 30 kb on chromosome 13q14, identified in 50% of chronic lymphocytic leukemia (CLL) [73], was one of the first indications of possible involvement of miRNAs in human tumorigenesis. After this first experimental evidence, the correlation between the genomic locations of a large number of miRNAs and cancer-associated genomic regions has been better identified [74].

From the functional and evolutive point of view, miRNAs represent for viruses an element of fundamental regulation not only of their genes but also of the host genes. Compared to genes coding for proteins, the genes coding for miRNAs are small; this characteristic is ideal for space-constrained restricted viral genomes. Moreover, their small size could facilitate rapid adaptation to new targets through small changes in the level of their nucleotide composition. In fact, a miRNA can have multiple targets and inhibit the expression of different genes simultaneously. These characteristics make miRNAs ideal candidates for control of host-pathogen interactions [26]. Increasing evidence suggests that EBV is a DNA tumor virus belonging to the human gamma-herpesvirus family, which is capable of establishing a latent infection mainly in human B lymphocytes and epithelial cells, and is associated with several human lymphoid and epithelial cell malignancies including EBVaGC [1–6]. Probably, the induction of tumors is not the main advantage of this virus but rather an accidental need to alter the cell cycle, preventing cell death, and evade the immune response [75]. In EBV-associated epithelial malignancies (e.g., NPC and EBVaGC) the gene products encoded by EBV play a crucial role at the beginning of the carcinogenesis process; therefore only few additional acquired genetic changes are required for the neoplastic transformation [10, 40, 41]. Specifically, in EBV-associated epithelial cancers, the latent genes (EBNA1, EBERs, and miR-BARTs) are intensely expressed in all cancer cells. Unlike EBNA1 and EBERs, miR-BARTs are expressed at high levels only in EBV-infected epithelial cancers, but not in EBV-transformed lymphocytes [56, 76, 77], pointing out their involvement in EBV-associated epithelial cancers. Interestingly, the expression of latency genes plays an important role in the initiation and neoplastic progression of gastric cancer by inducing strong antiapoptotic signals. For instance, EBER1 can confer an apoptotic-resistant phenotype by increasing the expression of insulin growth factor-1 (IGF-1), an autocrine growth factor which potentiates cell proliferation in EBVaGC [78]. During EBV infection of epithelial cells, these EBV-encoded viral regulatory RNAs may also modulate the host innate immune responses [79]. A recent study by Banerjee et al. [80] also suggested that EBERs could increase IL-6 expression and activate its downstream regulator STAT3, which was responsible for downregulation of the cell cycle inhibitors p21 and p27 in gastric carcinoma cells and associated cancer cell resistance. The same authors also demonstrated that EBERs could downregulate antimetastatic molecules, RhoGD1 and KAI-1, and activate the prometastatic molecules, pFAK and pPAK1, which induced cell migration. In addition, many EBV miRNAs also repress apoptosis by targeting the proapoptotic proteins p53-upregulated modulator of apoptosis (PUMA), Bcl-2 interacting mediator of cell death (BIM), and translocase of outer mitochondrial membrane 22 homolog (TOMM22), respectively [81–83]. Specifically, Choy and colleagues [81] have demonstrated that EBV miR-BART5 inhibits production of the proapoptotic protein PUMA by targeting mRNA which encodes the cellular factor PUMA. PUMA is one of the six members of the BH3-only group in the Bcl-2 family. The BH3-only proteins, the essential initiators of apoptosis, are responsible for controlling the release of cytochrome C from the mitochondrial intermembrane [84]. In EBV-infected carcinoma cells miR-BART5 depletion induces high levels of PUMA-mediated apoptosis, suggesting a crucial role of miR-BART5 in the inhibition of apoptosis both in EBV-infected epithelial cells and in EBV-transformed cells. Therefore, miR-BART5 may contribute to the survival of infected cells during the natural viral infection and influence the cell survival in virus-associated cancers. Another BH3-only group proapoptotic protein, Bim, has been reported to be a target of both EBV miR-BART4 and miR-BART15 [82, 85]. It is possible that this activity is linked to the observed inhibition of apoptosis by miRNAs of the BART cluster in the human gastric carcinoma cell line AGS. Interestingly, Bim's 3′-UTR is not responsive to any of the individual miR-BARTs in stable transfectants indicating possible cooperation of miR-BARTs in cluster 1 for Bim expression. Another potential target of miR-BART-16 is TOMM22, a protein forming part of a mitochondrial pore complex that stands as a receptor for the proapoptotic protein, Bcl-2-associated X (Bax) [83]. EBV can gain benefit from the repression of TOMM22 since siRNA-mediated knockdown of TOMM22 has been shown to inhibit the association of Bax protein to mitochondria, therefore preventing Bax-dependent apoptosis [86]. Other EBV miRNAs of the BART cluster variably expressed in EBVaGC tissue samples and cell lines are EBV-miR-BART1-3p, 5-5p, 7-3p, 15-3p, 19-3p, and 22-3p [87–89]. Recently, Shinozaki-Ushiku et al. [90] also reported that EBV-miR-BART4-5p exerts a crucial role in gastric tumorigenesis through modulation of apoptosis. Specifically, these authors demonstrated that reduction of apoptosis in clinical samples from EBVaGC patients was attributable to the expression of EBV-miR-BART4-5p which plays a partial role in suppressing proapoptotic protein Bid, also termed as the BH3 interacting domain death agonist. Another important finding has been recently reported by Kim et al. [91]. These authors suggested that miR-BART20-5p reduced Bcl-2-associated death promoter (BAD) expression in EBV-infected GC cells as opposed to EBV-negative GC cells by directly targeting 3′-UTR of BAD in order to promote host cell survival. Recently, Kanda et al. [92] also demonstrated that multiple EBV-encoded miRNAs contribute to EBVaGC by targeting N-myc downstream regulated gene 1 (NDRG1). This oncosuppressor protein was highly expressed in primary epithelial cells and significantly reduced in the BART(+) virus-infected epithelial cells, playing an important role in carcinogenesis and preventing the metastasis and invasion of gastric cancer cells. These data also implicate that NDRG1 could be utilized as a prognostic and/or diagnostic marker as well as a potential therapeutic target against gastric cancer [92, 93] (Table 1). Additional studies by Fu et al. [94] also demonstrated that EBV BHRF1 miRNAs can promote cell survival by interacting with several proapoptotic proteins such as Bcl-2 homologous antagonist/killer (Bak), Bcl-2-related ovarian death gene (Bod), Bcl-2-related ovarian killer (Bok), Bim, and Bcl-2-interacting killer (Bik) in various cell lines including gastric carcinoma cell lines. The same authors also indicate that BARF1 (Bam HI-A fragment rightward reading frame 1) might promote cell transformation by activating antiapoptotic protein Bcl-2 [94]. In addition to these effects, EBV infection may also affect host cell miRNA expression and exert effects on immune responses and oncogenesis. For instance, studies by Shinozaki et al. [95] have demonstrated downregulation of the cellular miR-200 family (e.g., miR-200a and miR-200b) in EBV-associated gastric carcinoma by repressing transcription of pri-miRNAs and by posttranscriptional dysregulation of the miRNA in EBVaGC compared to EBVnGC and adjacent mucosa. Specifically, these authors indicate that all the latency type I genes such as Bam HI-A rightward reading frame (BARF0), EBERs, EBNA1, and LMP2A have a synergetic effect on these processes and contribute to the downregulation of the mature miR-200 family and the subsequent upregulation of the E-cadherin transcription repressors, zinc finger E-box binding factor 1 (ZEB1) and ZEB2, resulting in the inhibition of E-cadherin expression and induction of the epithelial-to-mesenchymal transition (EMT) [95]. This is a pivotal stage in the process of carcinogenesis of EBV-associated gastric carcinoma. Another report recently published by Du and colleagues [96] also revealed that miR-141, a member of the miR-200 family, was downregulated in 80% of primary gastric cancer tissues showing its inhibitory effect on cell proliferation. A recent study by Marquitz et al. [97] suggests that, by infecting EBV-negative AGS gastric carcinoma cell lines with a recombinant EBV, a clear hallmark of transformation becomes apparent with an anchorage independent phenotype. Specifically, the authors showed that the cells have levels of BART miRNAs similar to EBV-positive gastric cancer. They also reported that EBNA1 overexpression in AGS gastric carcinoma cell lines significantly reduced miR-143 expression, which acts as tumor suppressor in several types of cancer [98–100]. More recently, Marquitz et al. [101] also indicate that EBV infection induces downregulation of host tumor suppressor miRNAs including the Let-7 family and the miR-200 family through a mechanism that is independent of latent protein expression. It seems that EBV miR-BART6 can repress the expression level of miRNA processing human DICER1 enzyme in EBV-infected cells compared to noninfected cells, playing an important role in the progression of EBV-associated tumors [102]. Therefore, EBV-encoded miRNAs may be an aetiological factor in cancer development in immunocompetent individuals that could bypass the requirement for viral protein expression and the consequential recognition by the immune system. Surprisingly, not all miR-BARTs inhibit apoptosis or promote cell growth. For example, Choi et al. [103] reported the tumor suppressive effect of miR-BART15 in gastric carcinogenesis. The authors demonstrated that miR-BART15 inhibited cell growth and induced early apoptosis in AGS gastric carcinoma cell in part by targeting the BRUCE gene which encodes the BRUCE protein, an inhibitor of apoptosis proteins (IAPs). In addition, miR-BART15 might also indirectly contribute to cancer development by inducing its proapoptotic activity in the adjacent immune cells by targeting BRUCE [103]. These results collectively suggest that some EBV-encoded miRNAs may regulate the expression of several key cancer-related proteins, including those involved in latency maintenance, immune suppression, and tumor promotion. Additional data would warrant a more solid conclusion on this issue.

## 6. Conclusions

Accumulating evidence suggests that miRNAs can be encoded not only by eukaryotes but also by certain viruses. The vast majority of viral miRNAs are encoded by the herpesviruses family including pathogens such as, EBV, herpes simplex viruses (HSVs), human cytomegalovirus (HCMV), Kaposi's sarcoma herpes virus (KSHV), and MHV 68. Specifically, EBV expresses multiple noncoding RNAs during all types of latency, including two clusters of miRNAs: BART miRNAs and BHRF1 miRNAs. EBV-transformed cells encode at least 44 mature viral miRNAs that target viral and cellular genes. Some viral miRNAs, including those which are analogs to host miRNAs and those which are virus-specific, seem to exert an important role in the establishment of a latent viral infection by suppression of an effective host immune response or through blockade of apoptotic processes in the infected cells [105, 106]. In all EBV-associated cancers, the viral infection also promotes important changes in the cellular miRNAs profile. It has been shown that many cell miRNAs in cell lines or in tumor tissues are deregulated upon EBV infection. The presently available information suggests that viral miRNAs also contribute to induction or maintenance of the transformed phenotype. In fact, latent infection with EBV is considered responsible for several malignancies, including a big amount of all gastric carcinomas. EBVaGC is characterized by unique clinical and pathologic features including male predominance, the presence of EBV genomes and EBV-encoded small RNA (EBER) in gastric carcinoma cell lines, and monoclonal proliferation of EBV-infected carcinoma cells [4, 9, 57, 104]. In addition, it also presents elevated levels of serum antibodies against EBV early antigen and EBV capsid antigen as well as a lymphoepithelioma-like histology and a relatively favourable prognosis [4, 9, 57, 104]. EBVaGC also shows abnormal hypermethylation of several tumor suppressor gene promoter regions, causing downregulation of their expression. In addition, EBVaGC is distinctive owing to the limited number of EBV latent genes expressed in cancer cells [96]. It is classified as latency type I because the expressed latent genes are restricted to BARF0, EBERs, EBNA1, and LMP2A, excluding EBNA2 or LMP1 which are essential for its transforming ability [107–109]. All four of these genes play an important role in the downregulation of mature miR-200 and the subsequent upregulation of transcription repressors ZEB1/ZEB2, resulting in the inhibition of E-cadherin expression and induction of the EMT, which is a crucial step in the carcinogenesis of EBVaGC. Notably, downregulation of mature miR-200 may be mediated by aberrant DNA methylation due to overexpression of DNMTs following EBV infection. EBER1 may also increase the expression of IGF-1, an autocrine growth factor which accelerates cell proliferation in EBVaGC [78]. Another important aspect underlined by Marquitz et al. [101] is that in several EBV-infected AGS gastric carcinoma cell lines a significant fraction of the changes in cellular expression likely reflect(s) the expression of the viral noncoding RNAs such as the BART RNAs and not the latent protein expression. These changes comprise a decrease in host tumor suppressor miRNAs levels and increased expression of viral miRNAs with putative oncogenic potential. However, the precise role of EBV in the multifactorial aetiology of gastric carcinoma is still not well known. In particular, it will be necessary to identify more targets of viral and deregulated cellular miRNAs. A general problem in EBV-encoded miRNAs research is the lack of an animal model. The advancement of humanized mice should facilitate the eventual development of EBV infection mouse models. Such models will ultimately be necessary to evaluate viral miRNAs as potential therapeutic cancer targets. They might be also used to evaluate different chemopreventive agents. In this respect, scientific evidence suggests that natural bioactive agents such as green tea catechins could be useful for the modulation of the epigenome [110] and the subsequent inhibition of viral infections and virus-associated malignancies [111–114]. Furthermore, green tea catechins may also inhibit chronic inflammation involved in viral oncogenesis by regulating the Nrf2 and NF-*κ*B signaling pathways [115–117].

## Figures and Tables

**Table 1:** List of main EBV-associated gastric carcinogenesis studies discussed in this review.

First author and year of publication	Study design	Paper number in references section	Summary of findings
Yau et al., 2014	Review	[10]	EBV infection contributes to gastric carcinogenesis through the expression of viral proteins and microRNAs as well as aberrant DNA methylation and histone modification and relative therapeutic implications.

Shinozaki-Ushiku et al., 2015	Review	[57]	In the present review latest findings on EBVaGC from clinicopathological and molecular perspectives were discussed to provide a better understanding of EBV involvement in gastric carcinogenesis. In addition to genetic and epigenetic changes, posttranscriptional gene expression regulation by cellular and/or EBV-derived microRNAs was also considered.

Shinozaki-Ushiku et al., 2015	Experimental research	[90]	Comprehensive profile of the expression of 44 known EBV miRNAs from EBV-associated gastric carcinoma patients was presented. Of several highly expressed EBV miRNAs, EBV-miR-BART4-5p plays a partial role in suppressing proapoptotic protein Bid, leading to reduced apoptosis. The present work provides novel insights into the roles played by EBV miRNAs in gastric carcinogenesis and identifies future potential therapeutic targets.

Kim et al., 2015	Experimental research	[91]	miR-BART20-5p contributes to the tumorigenesis initiation and/or maintenance of EBVaGC by directly targeting 3′-UTR of Bcl-2-associated death promoter (BAD) involved in cell proliferation and apoptosis. Inhibition of miR-BART20-5p can exert a therapeutic effect for this neoplasia.

Kanda et al., 2015	Experimental research	[92]	A causative relationship between BART miRNA expression and epithelial carcinogenesis in vivo was demonstrated. In particular, it was shown that NDRG1 protein, which is a putative target of BART miRNAs, can be used as an epithelial differentiation marker and a suppressor of metastasis.

Fu et al., 2013	Review	[94]	Potential mechanisms by which EBV contributes to its own latency and the formation tumors including EBVaGC were considered. Particularly, this review describes the interactions of EBV gene products including viral miRNAs and the Bcl-2 family members involved in cell death (apoptosis) and survival pathways. A better understanding of this complicated network of interactions could be of great importance for creating novel therapeutic strategies for EBV-associated diseases.

Tokunaga et al., 1993	Epidemiological research	[104]	EBV infection contributed significantly to gastric carcinogenesis in Japan. It occurred predominantly in males, especially in cancers of the upper and middle parts of the stomach, with greater cell type variation in men, suggesting that novel factors may play important causal roles in EBV-associated gastric carcinogenesis.
